# Survival and biomarkers for cachexia in non‐small cell lung cancer receiving immune checkpoint inhibitors

**DOI:** 10.1002/cam4.6549

**Published:** 2023-09-15

**Authors:** Daiki Murata, Koichi Azuma, Norikazu Matsuo, Kenta Murotani, Goushi Matama, Akihiko Kawahara, Tetsuro Sasada, Takaaki Tokito, Tomoaki Hoshino

**Affiliations:** ^1^ Division of Respirology, Neurology, and Rheumatology, Department of Internal Medicine Kurume University School of Medicine Fukuoka Japan; ^2^ Biostatistics Center Kurume University School of Medicine Fukuoka Japan; ^3^ Department of Diagnostic Pathology Kurume University Hospital Fukuoka Japan; ^4^ Cancer Vaccine and Immunotherapy Center and Division of Cancer Immunotherapy Kanagawa Cancer Center Research Institute Kanagawa Japan

**Keywords:** cachexia, chemokine, CRP, cytokine, NSCLC, PD‐1 inhibitor

## Abstract

**Background:**

The presence of cachexia negatively impacts the prognosis of patients with cancer. However, the mechanisms behind the development of cachexia and its prognostic impact on immunotherapy efficacy are not fully understood.

**Materials and Methods:**

We retrospectively screened patients with advanced or recurrent non‐small cell lung cancer (NSCLC) who received PD‐1/PD‐L1 inhibitor monotherapy. Among 183 patients, pre‐treatment plasma samples were available from 100 patients. We defined cancer cachexia as weight loss of at least 5% during the past 6 months or weight loss of at least 2% and BMI <20. We analyzed 75 soluble immune mediators in pre‐treatment plasma samples to explore the possible mechanisms behind the development of cancer cachexia. We also investigated whether cancer cachexia affects prognosis.

**Results:**

Among 100 patients, 35 had cancer cachexia. Logistic regression analysis identified ghrelin, c‐reactive protein (CRP), pentraxin‐3 (PTX‐3), and osteopontin (OPN) as factors associated with cachexia. Patients with cachexia had worse progression‐free survival (PFS) and overall survival (OS), although we did not detect statistically significant differences. Analyzing the soluble immune mediators associated with cachexia, the combination of cachexia and PTX‐3 or OPN expression levels was predictive for PFS and the combination of cachexia and CRP or OPN expression levels was predictive for OS.

**Conclusions:**

Pre‐treatment ghrelin, CRP, PTX‐3, and OPN may be associated with cachexia. Among patients with NSCLC who received PD‐1/L1 inhibitor monotherapy, those with cachexia had worse survival than those without cachexia. Larger studies will be required to confirm our data and better understand the mechanisms behind the development of cachexia.

## Graphical abstract

The mechanisms behind the development of cachexia and its prognostic impact on immunotherapy efficacy are not fully understood. Pre‐treatment ghrelin, CRP, PTX‐3, and OPN may be associated with cachexia in NSCLC receiving PD‐1 inhibitors. Patients with NSCLC and cachexia who received PD‐1/L1 monotherapy tended to have worse survival than those without cachexia irrespective of presence of ghrelin, CRP, PTX‐3, and OPN.

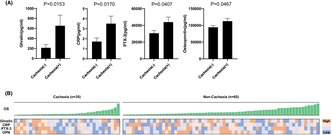

## 1 INTRODUCTION

Cachexia is a multifactorial syndrome defined by an ongoing loss of skeletal muscle mass (with or without loss of fat mass) that cannot be fully reversed by conventional nutritional support and that leads to progressive functional impairment.^1^ Cachexia is the most prevalent complication of cancer, affecting 50% of patients, and directly accounts for an estimated 22% of cancer deaths.^2^ Cancer cachexia results from a combination of increased systemic inflammation, excess energy expenditure, reduced energy intake, and elevated catabolism.^1^, ^2^, ^3^ The mechanisms behind the development of cachexia are not fully understood, and it is speculated that a complex network of multiple cytokines, such as IL‐6 and TNF‐α, is involved in the process.^2^, ^3^ Since the presence of cachexia in patients with cancer negatively affects the quality of life and decreases survival, it is important to appropriately manage cachexia.^1^, ^2^, ^3^, ^4^

Immune checkpoint inhibitors (ICIs) have demonstrated promising clinical outcomes for patients with advanced tumors in recent years.^2^, ^5^, ^6^, ^7^, ^8^, ^9^, ^10^ In non‐small cell lung cancer (NSCLC), a subset of patients treated with ICI have long‐duration tumor responses.^9^ However, ICI are known to be less beneficial in vulnerable patients, such as those with poor performance status (PS).^10^ Since cachexia is common in those with lung cancer and worsens the patient's condition, it is essential to determine the impact of cachexia on ICI treatment.^1^, ^2^, ^3^, ^4^ The immune status of cancer cachexia has been studied fairly extensively, but the role of various immune cells remains unclear.^1^, ^2^, ^3^, ^4^ Therefore, details on the mechanisms that inhibit the therapeutic efficacy of ICI also remain unknown.^2^, ^3^, ^4^ A comprehensive study of biomarkers associated with cancer cachexia might identify new therapeutic targets to improve the therapeutic efficacy of ICI.^2^, ^3^, ^4^

Hence, we measured a comprehensive number of soluble immune mediators and performed exploratory analysis in patients with NSCLC treated with ICI. In the present study, we analyzed soluble immune mediators in pre‐treatment plasma samples to explore the possible mechanisms behind the development of cancer cachexia. We also investigated whether cancer cachexia affects the survival of patients with NSCLC treated with programmed death protein 1 (PD‐1) or programmed death ligand 1 (PD‐L1) ICI. Furthermore, the soluble immune mediators associated with cancer cachexia were examined for their prognostic impact. The primary endpoint of our study was the detection of biomarkers associated with cancer cachexia. The secondary endpoint of the study was the evaluation of the prognostic impact of cancer cachexia and its biomarkers in patients with NSCLC treated with ICI.

## 2 MATERIALS AND METHODS

### 2.1 Patients and clinical analysis

We retrospectively screened patients with advanced or recurrent NSCLC who had received PD‐1/PD‐L1 inhibitor monotherapy at Kurume University Hospital between February 2016 and December 2020. Among 183 patients who received PD‐1/PD‐L1 inhibitor monotherapy, we analyzed 100 patients from whom a pre‐treatment plasma sample was available for analysis. All patients had pathologically confirmed NSCLC. PD‐L1 expression was assessed by the PD‐L1 IHC 22C3 pharm Dx assay on tumor cells in archived biopsy specimens. All patients were initiated on ICI treatment after confirming there were no symptoms of suspected infection, such as fever, cough or sputum. This study was performed in accordance with the provisions of the Declaration of Helsinki and was approved by the Institutional Review Board of Kurume University Hospital (IRB No 20100).

### 2.2 Definition of cancer cachexia

The definition of cancer cachexia used in this study was based on the diagnostic criteria in the international consensus proposed by Fearon et al. We defined patients as having cancer cachexia when they experienced more than 5% loss of stable body weight over the previous 6 months, or when they had a body mass index (BMI) under 20 kg/m^2^ and experienced ongoing weight loss of over 2%.^1^ We did not include skeletal muscle mass in our definition of cancer cachexia because measurement of skeletal muscle mass using software based on CT and MRI images is not available everywhere and cannot be used in routine medical practice.

### 2.3 Measurement of cachexia‐related factors in plasma

To detect which factors were associated with cancer cachexia, the levels of soluble immune mediators (e.g. cytokines, chemokines, growth factors) in pre‐treatment plasma samples were examined using a bead‐based multiplex assay and enzyme‐linked immune assay (ELISA). We chose soluble immune mediators based on previous reports that showed these factors were associated with cachexia or the tumor microenvironment.^2^, ^3^, ^11^, ^12^, ^13^ For example, we researched members of the IL family and TNF‐α, which are known to cause inflammation in cancer cachexia, and factors induced by these pro‐inflammatory cytokines, such as growth factors, matrix metalloproteinase family members, and other chemokines.^2^, ^3^, ^11^, ^12^, ^13^ For these assays, soluble immune mediators were measured in 100‐μl aliquots of two‐fold‐diluted plasma with a Bio‐Plex 200 system (Bio‐Rad Laboratories) according to the manufacturer's instructions. A total of 73 different soluble immune mediators were measured with analyte kits from Bio‐Rad Laboratories: 6Ckine, a proliferation‐inducing ligand (APRIL), B cell‐activating factor (BAFF), BCA‐1, chitinase 3‐like‐1, cutaneous T‐cell‐attracting chemokine (CTACK), epithelial‐neutrophil‐activating peptide (ENA)‐78, eotaxin, eotaxin‐2, eotaxin‐3, fractalkine, granulocyte chemotactic protein (GCP)‐2, Gro‐α, Gro‐β, granulocyte‐macrophage colony‐stimulating factor (GM‐CSF), glycoprotein (gp)130, I‐309, interleukin (IL)‐1β, IL‐2, IL‐4, IL‐6, IL‐8, IL‐10, IL‐11, IL‐12 (p40), IL‐12 (p70), IL‐16, IL‐19, IL‐20, IL‐22, IL‐26, IL‐27, IL‐29, IL‐32, IL‐34, IL‐35, IL‐6Rα, interferon (IFN)‐α2, IFN‐β, IFN‐γ, interferon‐gamma inducible Protein 10 kDa (IP‐10), interferon–inducible T cell alpha chemoattractant (I‐TAC), LIGHT, macrophage inflammatory protein (MIP)‐1α, MIP‐1δ, MIP‐3α, MIP‐3β, myeloid progenitor inhibitory factor (MPIF)‐1, monocyte chemoattractant protein (MCP)‐1, MCP‐2, MCP‐3, MCP‐4, macrophage‐derived chemokine (MDC), macrophage migration inhibitory factor (MIF), monokine induced by gamma interferon (MIG), matrix metalloproteinase (MMP)‐1, MMP‐2, MMP‐3, osteocalcin, osteopontin (OPN), pentraxin‐3 (PTX‐3), SCYB16, sCD30, sCD163, stromal cell derived factor (SDF)‐1 α + β, sTNF‐R1, sTNF‐R2, TARC, thymus expressed cytokine (TECK), tumor‐necrosis factor (TNF)‐α, thymic stromal lymphopoietin (TSLP), TNF‐related weak inducer of apoptosis (TWEAK), and vascular endothelial growth factor (VEGF). Plasma ghrelin levels were measured by a commercially available competitive enzyme‐linked immunoassay kit (Enzyme‐Linked Immunosorbent Assay, Organon Teknika). Plasma C‐reactive protein (CRP) levels were measured using an automatic clinical chemistry analyzer (LABOSPECT, Hitachi HighTech Co.). CRP levels were measured upon confirmation of no clinical findings suspicious for infectious disease, such as fever, cough, and sputum.

### 2.4 Statistical analysis

As this was an observational study, 100 participants were set as the target sample size to be included in this study through the participating facilities during the study period. Comparisons for categorical variables were evaluated using the chi‐squared or Fisher's exact tests. Progression‐free survival (PFS) and overall survival (OS) between groups were compared using the log‐rank test. A logistic regression analysis was employed to explore factors associated with cancer cachexia. Factors that were associated with cachexia in the logistic regression analysis were compared using the *t*‐test. For each biomarker associated with cachexia, the optimal cut‐off values for PFS and OS were determined using the web application Cutoff Finder.^14^ The optimal cut‐off was defined as the value resulting in the most significant split (log‐rank test). All tests were performed two‐sided, and differences were considered statistically significant at *p* < 0.05. In the logistic regression analyses, differences were considered a statistical trend at *p* < 0.1 to identify more factors that might be associated with cachexia. Statistical analyses were conducted using JMP pro version 16.0 statistical software (SAS Institute Inc.). The cut‐off date for analyses was August 31, 2022.

## 3 RESULTS

### 3.1 The association of cancer cachexia with patient characteristics

**FIGURE 1:**
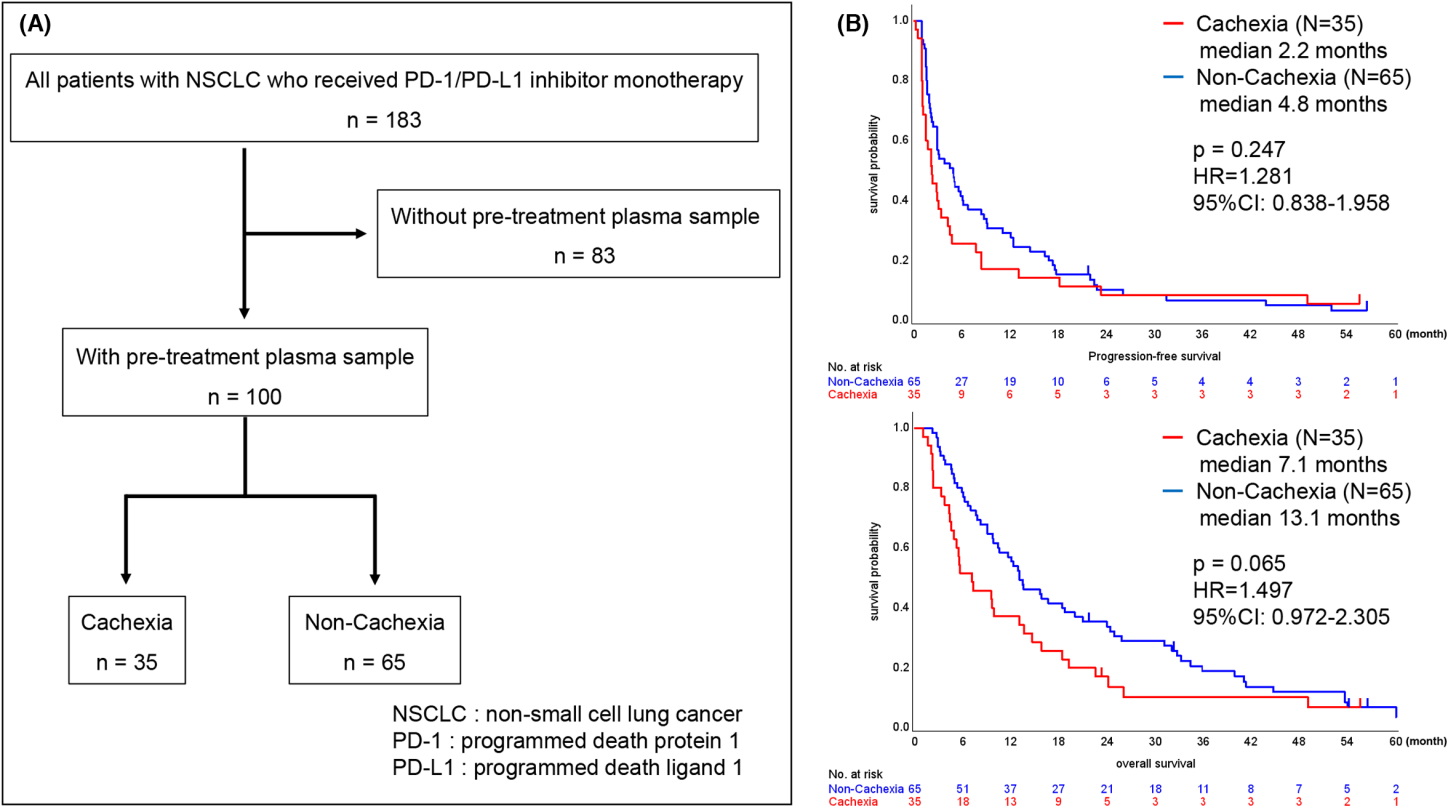
(A) Flow diagram of the study population. A total of 183 patients with non‐small cell lung cancer (NSCLC) who received programmed death protein 1 (PD‐1)/programmed death ligand 1 (PD‐L1) inhibitor monotherapy were screened, of whom 100 were eligible due to availability of pre‐treatment plasma samples. Of the 100 patients, 35 were diagnosed with cancer cachexia. (B) Kaplan–Meier survival curves of progression‐free survival (PFS) and overall survival (OS) for patients with and without cancer cachexia. (Upper) Median PFS was 2.2 months in the cachexia group and 4.8 months in the non‐cachexia group (hazard ratio (HR) = 1.281, 95% confidence interval (CI): 0.838–1.958, *p* = 0.247, log‐rank). (Lower) Median OS was 7.1 months in the cachexia group and 13.1 months in the non‐cachexia group (HR = 1.497, 95% CI: 0.972–2.305, *p* = 0.065, log‐rank).

Figure 1A shows a flow diagram of the study patients. Pre‐treatment plasma samples were available for 100 patients among patients with advanced or recurrent NSCLC treated with PD‐1/PD‐L1 inhibitor monotherapy between February 2016 and December 2020. Among these patients, 35 (35.0%) were diagnosed with cancer cachexia. Relevant patient characteristics in relation to the presence of cancer cachexia are summarized in Table 1. The presence of cancer cachexia was significantly associated with sex, PS, and smoking status (*p* = 0.001, 0.001, and 0.019, respectively).

**TABLE 1:** Comparisons of clinical characteristics between patients with NSCLC receiving PD‐1/PD‐L1 inhibitor monotherapy with and without cachexia.

Variable	With cachexia (*N* = 35)	Without cachexia (*N* = 65)	*p*
Age, median (range), years	74 (53–84)	70 (53–84)	0.073
Sex			**0.001**
Male	17 (48.6)	52 (80.0)	
Female	18 (51.4)	13 (20.0)	
ECOG performance status			**0.001**
0	6 (17.1)	38 (58.5)	
1	20 (57.1)	21 (32.3)	
2	8 (22.9)	4 (6.2)	
3	1 (2.9)	2 (3.1)	
Smoking status			**0.019**
Former or current	21 (60.0)	53 (81.5)	
Never	14 (40.0)	12 (18.5)	
Histology			0.494
Squamous	8 (22.9)	19 (29.2)	
Non‐squamous	27 (77.1)	46 (70.8)	
Driver mutation			0.095
EGFR or ALK	11 (31.4)	11 (16.9)	
Wild type	24 (68.6)	54 (83.1)	
Treatment line			0.566
First	7 (20.0)	17 (26.2)	
Second or later	28 (80.0)	48 (73.8)	
PD‐L1 TPS	(*N* = 31)	(*N* = 53)	0.781
<1%	11 (35.5)	15 (28.3)	
1%–49%	8 (25.8)	16 (30.2)	
≥50%	12 (38.7)	22 (41.5)	
PD‐1/PD‐L1 inhibitor			0.763
Nivolumab	22 (62.9)	36 (55.4)	
Pembrolizumab	11 (31.4)	24 (36.9)	
Atezolizumab	2 (5.7)	5 (7.7)	

*Note*: Data represent numbers (%) unless otherwise indicated.

Abbreviations: ALK, anaplastic lymphoma kinase; ECOG, Eastern Cooperative Oncology Group; EGFR, epidermal growth factor receptor; NSCLC, non‐small cell lung cancer; PD‐1, programmed death protein 1; PD‐L1, programmed death ligand 1; TPS, tumor proportion score.

The bold values in Table 1 indicate statistically significant with *p* < 0.05.

### 3.2 The association between cancer cachexia and survivals of PD‐1/PD‐L1 inhibitor monotherapy

The median PFS was 2.2 months in the cachexia group and 4.8 months in the non‐cachexia group (HR = 1.281, 95% CI: 0.838–1.958, *p* = 0.247, log‐rank). The median OS was 7.1 months in the cachexia group and 13.1 months in the non‐cachexia group (HR = 1.497, 95% CI: 0.972–2.305, *p* = 0.065, log‐rank). Figure 1B shows Kaplan–Meier curves for patients with and without cancer cachexia. There were no statistically significant differences in PFS and OS between the groups with and without cancer cachexia.

### 3.3 The association between plasma factors and cachexia

**FIGURE 2:**
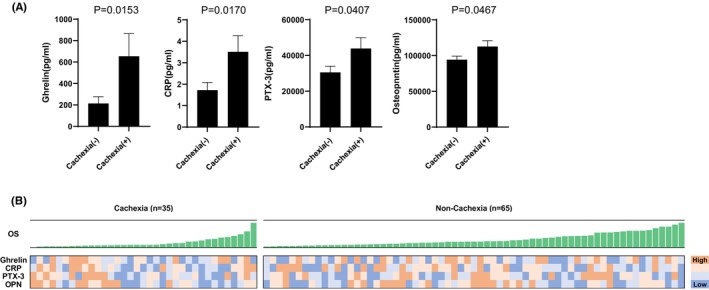
(A) Comparison of pre‐treatment plasma factor levels between patients with and without cachexia. The results of a *t*‐test for the following factors identified as associated with cancer cachexia by logistic regression analysis are shown: ghrelin (*p* = 0.0153), C‐reactive protein (CRP) (*p* = 0.0170), pentraxin (PTX)‐3 (*p* = 0.0407), osteopontin (OPN) (*p* = 0.0467). (B) Heatmap to show the levels of ghrelin, CRP, PTX‐3, and OPN in the cachexia and non‐cachexia groups. Colors show the levels of each soluble immune mediator in each patient.

To identify factors that might be associated with cancer cachexia, we analyzed pre‐treatment plasma samples for the concentrations of 75 different soluble immune mediators. Logistic regression analysis identified the following factors as associated with cancer cachexia: ghrelin (*p* = 0.041), CRP (*p* = 0.030), PTX‐3 (*p* = 0.045), and OPN (*p* = 0.052) (Table S1). Figure 2A shows the results of a *t*‐test revealing that the levels of ghrelin (*p* = 0.0153), CRP (*p* = 0.0170), PTX‐3 (*p* = 0.0407), and OPN (*p* = 0.0467) were significantly higher in patients with cancer cachexia than in those without cachexia. Figure 2B is a heatmap, which shows the levels of ghrelin, CRP, PTX‐3, and OPN in the cachexia and non‐cachexia groups.

### 3.4 The relation between plasma factors associated with cachexia and survival in response to PD‐1/PD‐L1 inhibitor monotherapy

We examined the prognostic impact of the four soluble immune mediators associated with cancer cachexia. Patients were classified into high and low groups based on the median levels of each immune mediator. In the cachexia group, the median levels were 173.1 pg/mL, 1.63 pg/mL, 51,255.8 pg/mL, and 110,010.3 pg/mL for ghrelin, CRP, PTX‐3, and OPN, respectively. As shown in Figure S1, there were no significant differences in PFS or OS between high and low groups for all of the assessed immune mediators.

**FIGURE 3:**
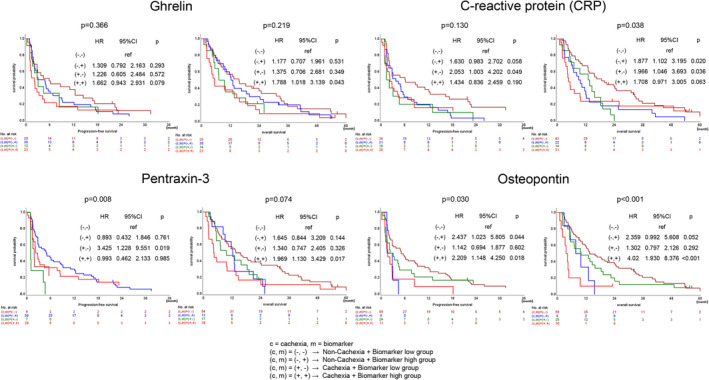
Kaplan–Meier survival curves for progression‐free survival (PFS) and overall survival (OS) in patients with and without cachexia. The groups were classified into high and low groups based on the optimal cut‐off values for each immune mediator.

In addition, we also examined the prognostic impact of groups with or without cachexia in combination with soluble immune mediators. Patients with and without cachexia were classified into high and low groups based on the optimal cut‐off values for each immune mediator. The optimal cut‐off values for each immune mediator were determined using the web application Cut‐off Finder.^14^ The optimal cut‐off values for PFS were 86.0 pg/mL, 0.66 pg/mL, 599.1 pg/mL, and 139,800 pg/mL for ghrelin, CRP, PTX‐3, and OPN, respectively. The optimal cut‐off values for OS were 104 pg/mL, 1.3 pg/mL, 49,940 pg/mL, and 141,300 pg/mL for ghrelin, CRP, PTX‐3, and OPN, respectively. Figure 3 shows Kaplan–Meier survival curves for the groups stratified by ghrelin, CRP, PTX‐3, and OPN levels in the cachexia and non‐cachexia groups. There were significant differences in PFS for the combination of cachexia and PTX‐3 (*p* = 0.008, log‐rank) or OPN (*p* = 0.030, log‐rank) expression levels. There were also significant differences in OS for the combination of cachexia and CRP (*p* = 0.038, log‐rank) or OPN (*p* < 0.001, log‐rank) expression levels, respectively.

## 4 DISCUSSION

Cancer cachexia results in a worsening of the patient's condition and is associated with a poor prognosis due to its high incidence and mortality.^1^, ^2^, ^4^ However, the mechanisms behind the development of cachexia and its impact on the efficacy of PD‐1/L1 inhibitors are not fully understood.^2^, ^4^, ^15^ Here, we analyzed soluble immune mediators in pre‐treatment plasma samples to explore the possible mechanisms behind cancer cachexia. We found that ghrelin, CRP, PTX‐3, and OPN expression levels may be associated with cachexia. In addition, we also investigated whether cancer cachexia affects the survival of patients with NSCLC treated with PD‐1/PD‐L1 inhibitors in clinical practice. We found that cachexia tended to result in worse survival in patients with NSCLC who received PD‐1/L1 inhibitor monotherapy, although the differences we detected were not statistically significant. Among the soluble immune mediators we detected that were associated with cachexia, the combination of cachexia and CRP, PTX‐3, or OPN expression levels were each associated with patient prognosis.

High ghrelin levels were significantly associated with cancer cachexia in this study. Previous studies have shown that patients with cachexia, as in the present study, tend to have higher ghrelin levels than those without cachexia.^3^ Ghrelin is a peptide hormone that is mainly produced in the stomach and presents as active and inactive forms.^16^, ^17^, ^18^, ^19^, ^20^ When activated, ghrelin stimulates appetite and suppresses energy expenditure,^16^, ^17^, ^18^, ^19^, ^20^ while the function of inactive ghrelin remains largely unknown.^17^, ^18^, ^20^ Inactive ghrelin was reported to reduce the proliferation of H345 small‐cell lung carcinoma cells in vitro, suggesting that inactive ghrelin may inhibit tumor progression.^17^, ^18^, ^20^, ^21^ Given the therapeutic effects of anamorelin on cachexia, ghrelin may play an important role in patients with cancer cachexia.^2^, ^16^, ^19^ However, ghrelin remains under study and its role is not well understood, especially regarding the functional differences between its active and inactive forms.^17^, ^18^, ^20^ We expect that cachexia may be treated more efficiently if we can more selectively manage the two forms of ghrelin.

We have shown that CRP and PTX3 may be associated with cachexia. These are members of the PTX family, which is associated with immune system activation and tumor progression.^22^, ^23^ Both CRP and PTX3 are highly sensitive factors reflecting systemic inflammation.^1^, ^22^, ^23^ The systemic inflammation caused by these cytokines directly affects muscle breakdown by increasing the loss of body cells in patients with cachexia.^2^, ^22^, ^23^ In patients with cachexia, systemic inflammation has been shown to alter host metabolism and promote increased glucocorticoid production, which causes loss of effector T‐cell infiltration, and abolishes responses to ICI treatment.^2^, ^24^ Therefore, it is suggested that suppressing tumor inflammation may improve the efficacy of immunotherapy in patients with cachexia. In fact, several drugs have been reported to inhibit tumor inflammation, including nonsteroidal anti‐inflammatory drugs and histamine antagonists.^22^, ^23^, ^25^, ^26^ CRP and PTX3 bind to the FCγ receptor and activate multiple downstream signaling pathways.^22^, ^23^ Interrupting some of these signaling pathways has potential to resolve therapeutic resistance to immunotherapy in cachexia. Nevertheless, since the inflammatory response is nonspecific, it is necessary to identify specific signals that may be therapeutic targets for cachexia.^1^, ^2^, ^22^, ^23^, ^25^ Cachexia and systemic inflammation are closely related, and further studies on the pathogenesis of systemic inflammation in patients with cachexia are warranted.

We also found that OPN may be associated with cancer cachexia. OPN is a phosphorylated multifunctional protein produced by tumor cells, fibroblasts, smooth muscle cells, endothelial cells, and immune cells.^27^ Through altering the tumor microenvironment, OPN is involved in tumor progression, chemotherapy resistance, and interference with immune function.^27^ OPN secreted by tumor‐associated macrophages upregulates PD‐L1 expression, which may result in chemotherapy resistance.^27^ Through the inhibition of CD8^+^ T‐cell activation and recruiting inhibitory macrophages, OPN also induces tumor immune tolerance.^27^ This immunomodulatory effect is assumed to reduce the efficacy of cancer immunotherapy.^27^ Therefore, OPN is thought to play a key role in treatment resistance in patients with cachexia, and management of OPN may improve the prognosis of patients with cachexia.^27^ Notably, neutralizing antibodies of OPN have been successful in the treatment of several diseases, such as osteoporosis, hepatitis, and arthritis.^27^ Future studies assessing the targeting OPN with these antibodies could determine the impact on the efficacy of chemotherapy and immunotherapy in patients with cancer cachexia.

In the present study, patients with cachexia who received immunotherapy had worse survival than those without cachexia, consistent with previous reports.^15^, ^28^ One reason for this may be related to the poor PS due to skeletal muscle wasting caused by cachexia.^1^, ^2^ Cachexia is known to be closely related to PS, as also observed in this study.^1^, ^2^ Immunotherapy is known to be less effective in patients with poor PS, suggesting a relationship with the poor survival of patients with cancer cachexia.^10^ We also found that among four biomarkers associated with cancer cachexia, the combinations of cachexia and CRP, PTX‐3, or OPN expression levels were associated with patient prognosis. Although confirmation of our data in further large‐scale studies is needed, given the small sample size in this study, our study showed that CRP, PTX‐3, and OPN expression levels might serve as prognostic factors in patients with cachexia. Since cancer cachexia is thought to reduce patient survival due to its complex mechanisms, further exploration of the network of cytokines underlying cachexia is warranted.

In summary, we have found that cachexia is associated with poor survival in patients with NSCLC who received PD‐1/L1 inhibitor monotherapy. Pre‐treatment ghrelin, CRP, PTX‐3, and OPN expression levels may be associated with cachexia. The combinations of cachexia and CRP, PTX‐3, or OPN might serve as predictors of survival in patients with NSCLC receiving immunotherapy. Nevertheless, our study had a few limitations. First, this study was a retrospective single‐center study. Second, the diagnosis of cachexia did not include sarcopenia. Since the mechanisms behind the development of cachexia are not fully understood, further studies based on our findings are needed to confirm the impact of the detected factors associated with cachexia on survival. Our findings provide a rationale for future clinical research on cancer cachexia as a therapeutic target for patients with NSCLC treated with ICI to improve therapy efficacy. Our study analyzed only pre‐treatment samples, but future studies measuring the levels of immune mediators pre‐ and post‐treatment may add more relevance to the data. Also, while we combined patients treated with PD‐1 and PD‐L1 ICI, future subgroup analysis studies assessing these immune mediators and their relationship to survival in response to either PD‐1 or PD‐L1 therapy may result in a better understanding of the pathogenesis. We collected the data retrospectively from patients treated with ICI monotherapy, but almost half of the patients could not be analyzed. It was not possible to increase the sample size due to the nature of this retrospective study. Thus, statistical tests in this study might have failed to detect significant differences due to the small sample size. Further large‐scale studies of patients with similar characteristics are needed to confirm our results.

## AUTHOR CONTRIBUTIONS

**Daiki Murata:** Conceptualization (lead); data curation (lead); formal analysis (equal); investigation (equal); methodology (equal); writing – original draft (lead); writing – review and editing (lead). **Koichi Azuma:** Conceptualization (lead); data curation (lead); investigation (lead); methodology (equal); project administration (lead); writing – original draft (lead); writing – review and editing (lead). **Norikazu Matsuo:** Data curation (equal); investigation (equal); methodology (equal). **Kenta Murotani:** Investigation (lead); methodology (lead). **Goushi Matama:** Investigation (equal); methodology (equal). **Akihiko Kawahara:** Data curation (equal); investigation (equal); methodology (equal). **Tetsuro Sasada:** Supervision (equal); writing – original draft (supporting); writing – review and editing (supporting). **Takaaki Tokito:** Data curation (equal); investigation (equal). **Tomoaki Hoshino:** Project administration (equal); writing – original draft (equal); writing – review and editing (equal).

## FUNDING INFORMATION

This study was supported by AMED (Grant Number 22ae0101076h0004).

## CONFLICT OF INTEREST STATEMENT

Koichi Azuma reports receiving personal fees from AstraZeneca, Bristol Myers Squibb, Chugai Pharmaceutical, MSD Oncology, and Ono Pharmaceutical, unrelated to the submitted work; Norikazu Matsuo reports receiving personal fees from Ono Pharmaceutical unrelated to the submitted work; Takaaki Tokito reports receiving personal fees from AstraZeneca, Boehringer Ingelheim, Chugai Pharmaceutical, and MSD, unrelated to the submitted work; Tetsuro Sasada reports receiving personal fees from Chugai and Bristol Myers Squibb, and research funds from Taiho and BrightPath Biotherapeutics, unrelated to the submitted work. The remaining authors have no conflicts of interest to disclose.

## ETHICS STATEMENT

All procedures involving human participants were performed in accordance with the ethical standards of the institutional and/or national research committee and with the 1964 Helsinki declaration and its later amendments or comparable ethical standards.

## INFORMED CONSENT

Informed consent was obtained from all individual participants included in the study.

## Supporting information


Figure S1.

Table S1.

## Data Availability

The data that support the findings of this study are available from the corresponding author upon reasonable request.
