# Supplementary figures and images for: Survival and biomarkers for cachexia in non‐small cell lung cancer receiving immune checkpoint inhibitors
Source: Cancer Med. 2023 Sep 15;12(19):19471–9. doi: 10.1002/cam4.6549 (PMC10587946; doi:10.1002/cam4.6549)

## Slide 1
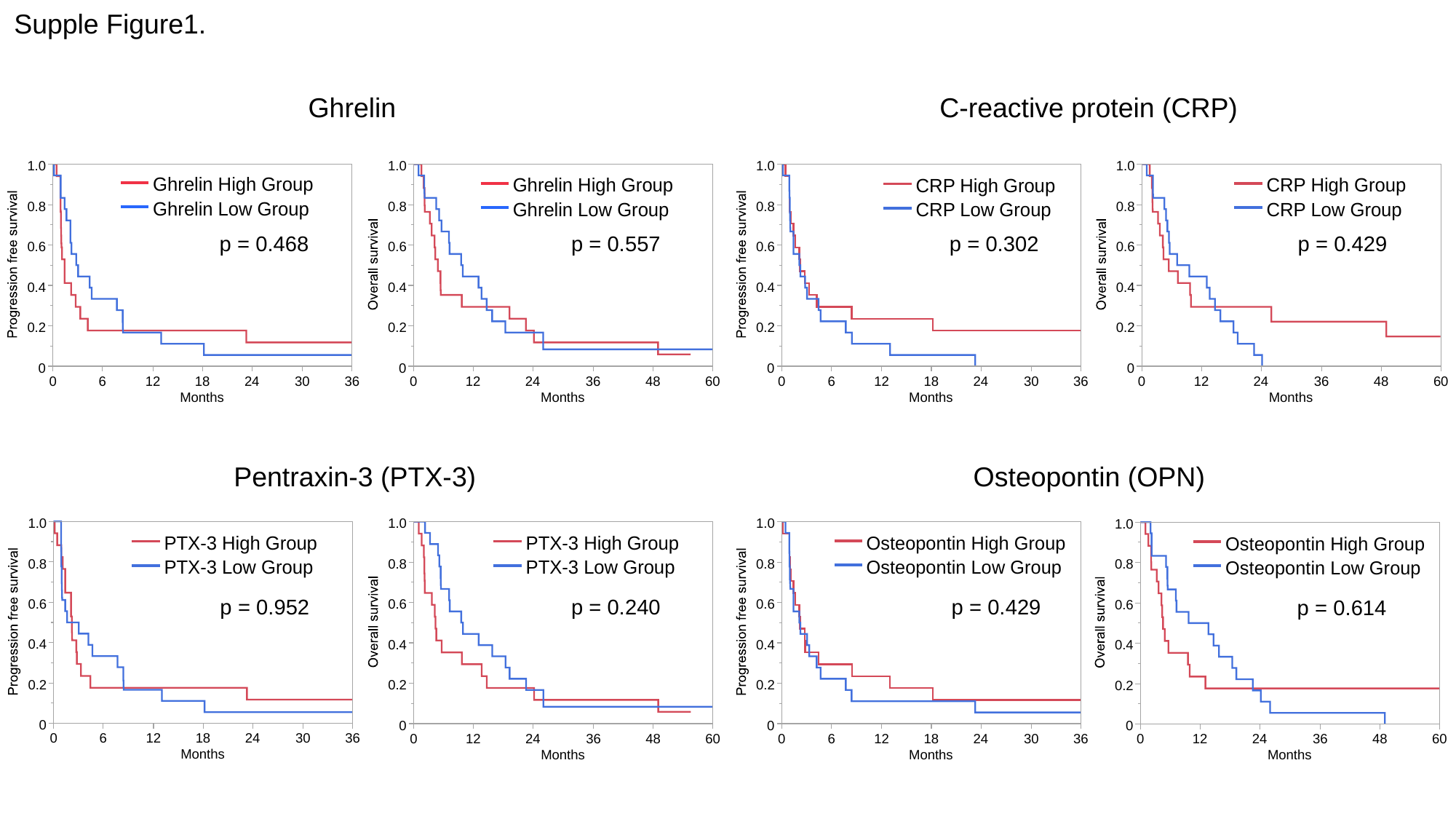

Supple Figure1.
Ghrelin
C-reactive protein (CRP)
p = 0.557
p = 0.429
p = 0.468
p = 0.302
Pentraxin-3 (PTX-3)
Osteopontin (OPN)
p = 0.429
p = 0.240
p = 0.952
p = 0.614

Supplement: Supplementary file 1 — Figure S1. [file CAM4-12-19471-s001.pptx]
