# Supplementary material for: Survival and biomarkers for cachexia in non‐small cell lung cancer receiving immune checkpoint inhibitors
Source: Cancer Med. 2023 Sep 15;12(19):19471–9. doi: 10.1002/cam4.6549 (PMC10587946; doi:10.1002/cam4.6549)
Supplement: Supplementary file 2 — Table S1. [file CAM4-12-19471-s002.docx]

**Supplemental Table 1. Logistic regression analysis of associations between immune mediators and cancer cachexia**

| Variable | Odds Ratio | 95%CI | P-value |
| --- | --- | --- | --- |
| Ghrelin | 1.001 | 1.000-1.001 | 0.041 |
| CRP | 1.152 | 1.014-1.308 | 0.030 |
| IL-1β | 1.005 | 0.766-1.319 | 0.970 |
| IL-2 | 0.999 | 0.995-1.003 | 0.562 |
| IL-4 | 0.983 | 0.955-1.012 | 0.248 |
| IL-6 | 1.007 | 0.990-1.024 | 0.421 |
| IL-8/CXCL8 | 1.001 | 0.999-1.003 | 0.471 |
| IL-10 | 0.997 | 0.988-1.006 | 0.459 |
| IL-11 | 1.001 | 0.989-1.014 | 0.816 |
| IL-12(p40) | 1.000 | 0.998-1.001 | 0.589 |
| IL-12(p70) | 0.997 | 0.984-1.010 | 0.627 |
| IL-16 | 0.998 | 0.994-1.003 | 0.490 |
| IL-19 | 1.000 | 0.994-1.006 | 0.928 |
| IL-20 | 0.998 | 0.993-1.004 | 0.558 |
| IL-22 | 1.000 | 1.000-1.000 | 0.452 |
| IL-26 | 1.000 | 1.000-1.000 | 0.543 |
| IL-27(p28) | 0.999 | 0.998-1.001 | 0.512 |
| IL-29/IFN-α1 | 1.000 | 0.998-1.001 | 0.628 |
| IL-32 | 1.000 | 0.999-1.001 | 0.572 |
| IL-34 | 1.000 | 1.000-1.000 | 0.653 |
| IL-35 | 1.000 | 0.999-1.000 | 0.602 |
| IFN-α2 | 0.999 | 0.997-1.002 | 0.559 |
| IFN-β | 1.000 | 0.996-1.005 | 0.899 |
| IFN-γ | 0.998 | 0.990-1.006 | 0.604 |
| TNF-α | 0.997 | 0.978-1.016 | 0.743 |
| GM-CSF | 0.987 | 0.930-1.048 | 0.670 |
| 6Ckine/CCL21 | 1.000 | 1.000-1.000 | 0.385 |
| BCA-1/CXCL13 | 1.012 | 0.959-1.068 | 0.656 |
| CTACK/CCL27 | 1.000 | 0.999-1.002 | 0.635 |
| ENA-78/CXCL5 | 0.999 | 0.997-1.001 | 0.345 |
| Eotaxin/CCL11 | 0.962 | 0.911-1.015 | 0.157 |
| Eotaxin-2/CCL24 | 1.007 | 0.992-1.022 | 0.385 |
| Eotaxin-3/CCL26 | 0.975 | 0.933-1.019 | 0.266 |
| Fractalkine/CX3CL1 | 1.003 | 0.998-1.007 | 0.229 |
| GCP-2/CXCL6 | 0.970 | 0.881-1.068 | 0.531 |
| GRO-α/CXCL1 | 0.998 | 0.991-1.005 | 0.561 |
| GRO-β/CXCL2 | 1.000 | 0.984-1.015 | 0.970 |
| IP-10/CXCL10 | 1.003 | 0.996-1.011 | 0.408 |
| I-TAC/CXCL11 | 1.001 | 0.941-1.066 | 0.965 |
| MIP-1α/CCL3 | 1.000 | 0.991-1.009 | 0.975 |
| MIP-1δ/CCL15 | 1.000 | 1.000-1.000 | 0.883 |
| MIP-3α/CCL20 | 1.066 | 0.962-1.181 | 0.224 |
| MIP-3β/CCL19 | 0.995 | 0.984-1.006 | 0.347 |
| MPIF-1/CCL23 | 1.002 | 0.999-1.004 | 0.215 |
| SDF-1α+β/CXCL12 | 1.000 | 0.999-1.001 | 0.541 |
| TARC/CCL17 | 0.990 | 0.962-1.019 | 0.505 |
| TECK/CCL25 | 0.999 | 0.997-1.001 | 0.192 |
| I-309/CCL1 | 0.982 | 0.942-1.024 | 0.392 |
| MCP-1/CCL2 | 0.995 | 0.986-1.003 | 0.210 |
| MCP-2/CCL8 | 0.952 | 0.854-1.061 | 0.373 |
| MCP-3/CCL7 | 0.995 | 0.984-1.007 | 0.442 |
| MCP-4/CCL13 | 0.964 | 0.920-1.011 | 0.130 |
| MDC/CCL22 | 1.000 | 0.997-1.003 | 0.874 |
| MIF | 1.000 | 1.000-1.000 | 0.250 |
| MIG/CXCL9 | 1.001 | 0.998-1.003 | 0.617 |
| APRIL/TNFSF13 | 1.000 | 1.000-1.000 | 0.740 |
| BAFF/TNFSF13B | 1.000 | 1.000-1.000 | 0.192 |
| sCD30/TNFRSF8 | 1.000 | 1.000-1.000 | 0.306 |
| sCD163 | 1.000 | 1.000-1.000 | 0.900 |
| Chitinase 3-like 1 | 1.000 | 1.000-1.000 | 0.849 |
| gp130/sIL-6Rβ | 1.000 | 1.000-1.000 | 0.653 |
| IL-6Rα | 1.000 | 1.000-1.000 | 0.401 |
| LIGHT/TNFSF14 | 1.000 | 0.999-1.000 | 0.540 |
| MMP-1 | 1.000 | 1.000-1.000 | 0.569 |
| MMP-2 | 1.000 | 1.000-1.000 | 0.786 |
| MMP-3 | 1.000 | 1.000-1.000 | 0.997 |
| Osteocalcin | 1.000 | 1.000-1.000 | 0.243 |
| Osteopontin | 1.000 | 1.000-1.000 | 0.052 |
| Pentraxin-3 | 1.000 | 1.000-1.000 | 0.045 |
| SCYB16/CXCL16 | 1.000 | 0.998-1.003 | 0.763 |
| sTNF-R1 | 1.000 | 1.000-1.000 | 0.286 |
| sTNF-R2 | 1.000 | 1.000-1.000 | 0.210 |
| TSLP | 0.996 | 0.988-1.005 | 0.415 |
| TWEAK/TNFSF12 | 0.999 | 0.997-1.000 | 0.164 |
| VEGF | 1.015 | 0.995-1.034 | 0.135 |

95% CI :95% Confidence interval

APRIL: a proliferation-inducing ligand, BAFF: B cell-activating factor, BCA: B cell-attracting chemokine, CCL: C-C motif chemokine ligand, CRP: C-reactive protein, CTACK: cutaneous T cell-attracting chemokine, CX3CL1: C-X3-C motif chemokine ligand 1, CXCL: chemokine (C-X-C motif) ligand, ENA: epithelial neutrophil activating peptide, GCP: granulocyte chemotactic protein, GM-CSF: granulocyte macrophage-colony stimulating factor, gp: glycoprotein, GRO: growth-related oncogene, ICI: Immune-checkpoint inhibitors, IFN: interferon, IL: interleukin, ILAs: interstitial lung abnormalities, ILD: interstitial lung disease, IP: interferon gamma-induced protein, I-TAC: interferon-gamma-inducible T-cell alpha chemoattractant, MCP: monocyte chemotactic protein, MDC: macrophage-derived chemokine, MIF: macrophage migration inhibitory factor, MIG: monokine-induced by gamma interferon, MIP: macrophage inflammatory protein, MMP: matrix metalloproteinase, MPIF: myeloid progenitor inhibitory factor, sCD: soluble cluster of differentiation, SDF: stromal cell derived factor, TARC: thymus and activation-regulated chemokine, TECK: thymus-expressed chemokine, TNF: tumor necrosis factor, TNFRSF: tumor necrosis factor receptor superfamily, TNFSF: tumor necrosis factor superfamily, TSLP: thymic stromal lymphopoietin, TWEAK: tumor necrosis factor-like weak inducer of apoptosis, VEGF: vascular endothelial growth factor.
